# The Genetics of Alzheimer's Disease

**DOI:** 10.6064/2012/246210

**Published:** 2012-12-31

**Authors:** Robert C. Barber

**Affiliations:** Department of Pharmacology and Neuroscience, University of North Texas Health Science Center, 3500 Camp Bowie Boulevard, Fort Worth, TX 76107, USA

## Abstract

Alzheimer's disease is a progressive, neurodegenerative disease that represents a growing global health crisis. Two major forms of the disease exist: early onset (familial) and late onset (sporadic). Early onset Alzheimer's is rare, accounting for less than 5% of disease burden. It is inherited in Mendelian dominant fashion and is caused by mutations in three genes (*APP*, *PSEN1*, and *PSEN2*). Late onset Alzheimer's is common among individuals over 65 years of age. Heritability of this form of the disease is high (79%), but the etiology is driven by a combination of genetic and environmental factors. A large number of genes have been implicated in the development of late onset Alzheimer's. Examples that have been confirmed by multiple studies include *ABCA7*, *APOE*, *BIN1*, *CD2AP*, *CD33*, *CLU*, *CR1*, *EPHA1*, *MS4A4A/MS4A4E/MS4A6E*, *PICALM*, and *SORL1*. Despite tremendous progress over the past three decades, roughly half of the heritability for the late onset of the disease remains unidentified. Finding the remaining genetic factors that contribute to the development of late onset Alzheimer's disease holds the potential to provide novel targets for treatment and prevention, leading to the development of effective strategies to combat this devastating disease.

## 1. Introduction

 The disease that would, upon the suggestion of his colleague Dr. Emil Kraepelin, eventually bear his name was first described by Aloysius “Alois” Alzheimer in 1907, based upon his observations and treatment of a 51-year-old patient, August “D” [1]. In addition to short-term memory loss, symptoms included unusual behavior and the neuropathological characteristics which have become hallmarks of the disease [1]. These features include extracellular plaques formed from cleaved amyloid precursor protein (APP) and intracellular tangles of hyperphosphorylated microtubule associated protein tau (MAPT) [2–4]. APP is an integral membrane protein with wide expression throughout the body, which is concentrated in neuronal synapses [5]. Although the primary function of APP is not fully understood, it has been implicated in neurite extension and synaptic plasticity. The tau protein is expressed primarily in neurons, where it stabilizes microtubules that are responsible for axonal transport [6].

Alzheimer's disease is a progressive, neurodegenerative disease that is ultimately and invariably fatal, unless another cause of death intervenes. Although the clinical course is heterogeneous, there are a number of common features in addition to the characteristic extracellular plaques and neurofibulary tangles. Early symptoms are often mistaken as part of normal aging, or manifestations of stress [7]. The most common symptom that is first noticed is a loss of short-term memory. As the disease progresses, symptoms may include aggression, irritability, confusion, and language problems, as well as loss of long-term memory. In later stages of the disease, patients become withdrawn and ultimately are completely unable to care for themselves [7]. Neuropathological changes include loss of neurons and synapses in the cerebral cortex and certain subcortical regions. This loss results in gross atrophy of the affected regions, including degeneration in the temporal and parietal lobes, as well as parts of the frontal cortex and cingulate gyrus [8]. MRI and PET analyses have documented atrophy of specific brain regions, particularly the hippocampus, as individuals progress from mild cognitive impairment to Alzheimer's disease [9, 10].

 Alzheimer's disease is the most common form of age-related neurodegenerative dementia and one of the most serious health problems in the industrialized world. More than 35 million individuals suffer from dementia globally, and the majority of these are diagnosed with Alzheimer's disease. Alzheimer's disease is also a major public health problem in the United States. The Alzheimer's Association reports that in 2011, 5.4 million individuals were diagnosed with Alzheimer's in the US, with an estimated prevalence ranging from 6%–12% of individuals aged 65 years and older [11]. This figure is expected to increase to over 10 million over the next 25 years, as individuals born between 1946 and 1964 begin reaching the age where they are at risk of developing the disease. Alzheimer's is the 6th leading cause of death in America and the 5th leading cause of death for those over 65 [11]. Currently available therapies provide short-term symptomatic relief but do not slow disease progression. In order for novel therapeutic approaches to be created, greater knowledge regarding the underlying etiology of the disease is needed. The financial costs of Alzheimer's currently exceed $183 billion annually in the US, and an additional $210 billion worth of unpaid care is provided by friends and family members of patients [11]. 

 There are two types of Alzheimer's disease: familial (also known as early onset) and sporadic (also known as late onset). Familial Alzheimer's disease is inherited in a Mendelian fashion, with little influence from the environment. In contrast, while genetics play a large role in the development and expression of late onset Alzheimer's disease, nongenetic factors are also very important. To date, there is a large literature documenting that obesity, diabetes, cardiovascular disease, and related factors, as well as cerebral and systemic inflammation increase the risk for late onset Alzheimer's disease, as well as lower the age at which symptoms first appear.

## 2. Familial Alzheimer's Disease

 Familial Alzheimer's disease (FAD) is expressed as a Mendelian trait with dominant inheritance. FAD is relatively rare, accounting for less than 5% of the total Alzheimer's disease burden [11, 14]. The Alzheimer's Association estimates that in 2011, approximately 200,000 individuals were afflicted with FAD in the US. A diagnosis is typically made when patients are in their fifties or sixties. However, there are instances of FAD onset at a much younger age. In one famous and particularly tragic example within an extended Columbian family, disease onset occurs in the mid-forties and as early as the early thirties [14]. The mutation responsible in this Columbian family (Presenilin 1 E280A) is known in other FAD families with a more typical age at onset.

 To date, more than 160 highly penetrant but rare mutations have been described in three genes (amyloid precursor protein, presenilin 1, and presenilin 2) that cause familial Alzheimer's disease (Table 1). Each of these genes will be discussed later.

### 2.1. Amyloid Precursor Protein (APP)

 The function of APP is not completely understood. However, it has been implicated in neuronal development and synaptic formation and repair and has been shown to be upregulated after neuronal injury. The gene encoding APP is located on chromosome 21q21.3. The gene consists of 19 exons and covers approximately 240 kilobases of DNA. The full-length APP matures in the Golgi and endoplasmic reticulum, prior to being inserted into the plasma membrane. The resulting protein is between 365 and 770 amino acids in length. It is expressed in numerous tissues, and at least eight isoforms are created by alternate splicing of exons 1–13, 13a, and 14–18. The most abundant isoforms are APP695 (exons 1–6, 9–18), APP751 (exons 1–7, 9–18), and APP770 (exons 1–18). All of the transcripts produce a multidomain protein with a single membrane-spanning region. APP751 and APP770 differ from APP695 in that they contain exon seven, which encodes a serine protease inhibitor domain. APP695 is the predominant isoform in neuronal tissues, whereas APP751 is the predominant isoform elsewhere. Full-length APP is processed into a number of fragments through a series of proteolytic cleavages by alpha-, beta- and gamma-secretase. Cleavage by alpha-secretase creates a fragment that is not associated with plaques or Alzheimer's. However, cleavage by beta-, and gamma-secretases creates beta-amyloid, which is encoded by exons 16 and 17 and is 39 to 42 amino acids in length. It is the beta-amyloid fragment that forms the extracellular plaques that are the hallmarks of Alzheimer's disease.

 APP was initially implicated in the etiology of Alzheimer's disease by a number of facts. First, the primary component of the extracellular plaques that characterize the disease is the amyloid protein. Tanzi and colleagues and Robakis et al. demonstrated in 1987 that the form of APP that is localized in plaques was derived from a larger protein that is encoded by a gene located on chromosome 21 [15, 16]. Second, chromosome 21 is duplicated in Down's syndrome patients, who typically develop Alzheimer's in the fifth decade of life. An extra copy of the APP gene is duplicated along with the other chromosome 21 genes in Down's syndrome, which further implicated the APP gene in Alzheimer's pathology.

**Figure 1:**
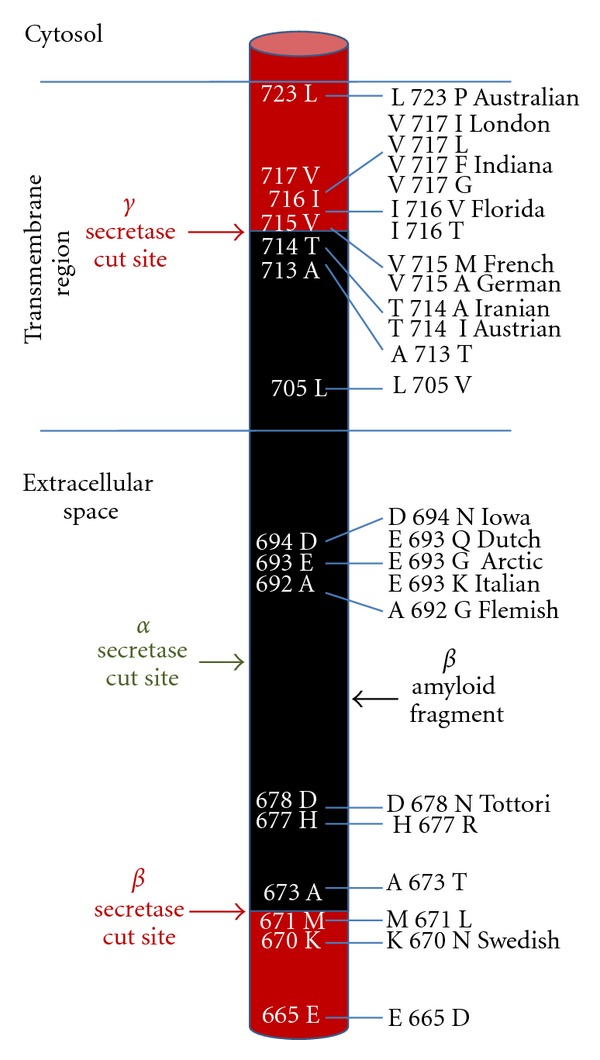
Processing of the amyloid precursor protein (APP). The precursor protein is acted upon by either alpha- or beta-secretase, followed by cleavage by gamma-secretase. Cleavage by beta-secretase allows formation of beta-amyloid (shown in black) by gamma-secretase, while alpha-secretase prevents beta-amyloid formation. Locations of the alpha-, beta-, and gamma-secretase cut sites are shown, along with APP mutations and the names that are associated with certain mutations.

 The beta-amyloid that forms the core of amyloid plaques consists of aggregates of a 4.2 kDa polypeptide and is the same in Alzheimer's and older Down's syndrome patients. The existence of this fragment, along with the details of how it is produced from the full-length protein, was resolved in a series of experiments in the mid-eighties by Tanzi, Masters, Robakis, Kang, Glenner, and Wong (Figure 1). These findings laid the groundwork for the amyloid cascade hypothesis of Alzheimer's disease, which postulates that production of beta-amyloid 42 fragments accumulates in the brain with age [17]. Either the resulting plaques, or perhaps the soluble oligomers, are neurotoxic and precipitate hyperphosphorylation of the tau protein, as well as other events that occur downstream in the disease process, including pruning of the dendritic tree and synaptic dysfunction. These changes result in neuronal loss, dementia, and ultimately death.

Lustbader et al. demonstrated that beta-amyloid interacted with amyloid beta-binding alcohol dehydrogenase (ABAD) to induce mitochondrial toxicity in Alzheimer's disease patients and in transgenic mice [18]. Crystallography showed that binding of beta-amyloid by ABAD deformed the nicotinamide adenine dinucleotide (NAD) binding site and prevented activation. Furthermore, inhibiting interactions between beta-amyloid and ABAD suppressed the generation of free radicals and beta-amyloid induced apoptosis within neurons. 

Linkage was detected between FAD and markers on chromosome 21 near the APP gene in 1987 [19], but subsequent studies failed to detect this association in families with late onset Alzheimer's disease [20]. An association between a missense mutation in the APP gene and FAD in a large kindred was detected in 2006 by Goate [21]. However, nonallelic heterogeneity was detected in this study as well as many others, implicating additional genes in the pathogenesis of familial Alzheimer's disease [20–22].

 APP mutations account for a minority of FAD cases but have also been shown to cause cerebral amyloid angiopathy. In a multicenter study of familial and sporadic Alzheimer's disease, Tanzi and colleagues concluded that mutations in the APP gene account for a small portion of FAD cases [23]. In a similar study, Kamino et al. also found that APP mutations account for a small fraction of FAD [24]. In affected members of 2 families with early onset Alzheimer's disease-1, Goate et al. identified a heterozygous mutation in the APP gene (V717I) [25]. Raux and others identified mutations in the APP gene among affected members in five of 31 families with FAD [26]. Combined with the results of earlier studies, the authors estimated that 16% of FAD is attributable to mutations in the APP gene [26]. 

 There have been extensive studies of the effects of APP mutations, particularly those occurring at amino acid 717. Suzuki et al. reported that 3 such mutations (V717I, V717F, and V717G) were consistently associated with a 1.5- to 1.9-fold increase in the generation of longer beta-amyloid fragments, which formed insoluble amyloid fibrils more rapidly than shorter fragments [27]. Yamatsuji and colleagues demonstrated that expression of the cytoplasmic domain of any of the mutations at amino acid 717 (V717I, V717F, and V717G) induced G protein-mediated nucleosomal DNA fragmentation in cultured neuronal cells [28]. 

 In a notable recent discovery, Jonsson and colleagues discovered a rare mutation (A673T) in the APP gene that was protective against Alzheimer's disease [29]. The mutation was discovered in a search for rare coding variants within whole-genome sequence data from 1,795 Icelanders. This A673T mutation was also found to be protective against cognitive decline among elderly without Alzheimer's disease. This substitution was adjacent to the beta-secretase site in APP, and resulted in a 40% reduction in the formation of amyloidogenic peptides.

### 2.2. Presenilins 1 and 2 (PSEN1 and PSEN2)

 The presenilins are important determinants of gamma-secretase activity and are responsible for proteolytic cleavage of the amyloid precursor protein (APP) and NOTCH receptor proteins [12]. Gamma-secretase is a multimeric protein complex consisting of PSEN1 or PSEN2, nicastrin, and APH1 [12]. All mutations in *PSEN1* increase cleavage of the amyloid precursor protein by gamma-secretase, thereby increasing the production of the beta-amyloid 42 fragment [13, 30–33]. Presenilin 1 is an evolutionarily conserved membrane protein that is present in *Drosophila* [34] and *C. elegans* [35]. The *PSEN1* gene, which is located on chromosome 14q24.2, was identified as an Alzheimer's disease locus in 1995 by positional cloning [36]. The *PSEN2* gene is located on chromosome 1q42.13 and has significant sequence homology and very similar structural organization to *PSEN1* (approximately 60% overall identity). In fact, it was the high degree of sequence identity between the two genes that led to the identification of *PSEN2* during the cloning of *PSEN1* [37]. However, it seems that the presenilin genes encode proteins with overlapping, but distinct functions. For example, mutations in both presenilin genes result in increased beta-amyloid [30, 32], yet functional *PSEN2* does not rescue APP or NOTCH signaling defects observed in *PSEN1* null animals [38]. 

 Evidence that presenilin genes were involved in gamma-secretase activity and hence in amyloid precursor protein processing was first obtained in 2000 when Li and colleagues suggested that PSEN1 and PSEN2 comprised the active site of gamma-secretase [39]. In their experiments, gamma-secretase activity coeluted with PSEN1 on gel exclusion chromatography. In addition, they observed that an anti-PSEN1 antibody immunoprecipitated active gamma-secretase. In the same year, Kopan and Goate determined that PSEN1 and PSEN2 both appear to be active gamma-secretase sites that reside in different complexes [40]. The authors proposed that regulation of cleavage may depend on the specific profile of proteins present in the multimeric gamma-secretase complex [40]. Lee et al. reported that nicastrin and presenilin heterodimers associate with APH1A and APH1B to form the gamma-secretase complex that is responsible for the proteolytic cleavage of many membrane proteins, including APP and NOTCH [12].

 Cai and colleagues reported that PSEN1 binds and recruits phospholipase D1 (PLD1) to the trans-Golgi network (TGN). Furthermore, expression of PLD1 in mouse neuroblastoma (N2a) cells was found to be inversely correlated with gamma-secretase-mediated beta-amyloid generation [41]. Additional studies by the same lab showed that overexpression of catalytically active PLD1 promoted generation of beta-amyloid-containing vesicles from the TGN [42]. Taken together, these observations showed that PLD1 regulates intracellular trafficking of beta-amyloid, distinct from its effect on gamma-secretase activity.

 Activity of PSEN1 and PSEN2 is essential for the formation of beta-amyloid 42. Transgenic mice that overexpressed mutant presenilin-1 showed an increase in beta-amyloid 42. However, mice overexpressing a wildtype version of presenilin-1 did not show a similar increase [31]. These results suggested that mutations in presenilin cause Alzheimer's disease through a deleterious gain of function that increases the amount of beta-amyloid 42 that is deposited in the brain. Support for this model of Alzheimer's etiology was provided by Davis et al. who showed that amyloid deposition was equivalent in the brains of wildtype mice and those with a loss of functional *Psen1* allele [43]. Finally, Qian and colleagues showed that the brains of mice carrying the human *PSEN1* A246E mutation showed increased levels of beta-amyloid 42 [44]. Citron and colleagues noted that several lines of evidence strongly support the conclusion that progressive cerebral deposition of beta-amyloid protein is a seminal event in familial Alzheimer's disease pathogenesis [32]. Both in vitro and in vivo data demonstrate that FAD-linked presenilin mutations directly or indirectly alter the level of gamma- but not alpha- or beta-secretase, resulting in increased production of beta-amyloid 42 which may lead to cerebral beta-amyloidosis and AD [32].

 Nearly 200 variant *PSEN1* alleles have been detected (http://www.molgen.ua.ac.be/ADMutations). The vast majority of these are missense mutations, which alter the protein sequence by a single amino acid. Although a few splice-site mutations and insertion-deletion variants have also been discovered, no nonsense mutations, which result in a truncated and nonfunctional protein, have been observed to cause Alzheimer's disease [45, 46].

 Relative to *PSEN1*, a much smaller number of mutations (approximately two dozens) have been observed to date in *PSEN2* in families segregating FAD (http://www.molgen.ua.ac.be/ADMutations). Similar to *PSEN1*, these are missense mutations with a deleterious gain of function effect. The range in age at onset is much more variable within families segregating *PSEN2* mutations, relative to those segregating disease alleles in *APP* or *PSEN1* [47–50]. In families that develop FAD due to *PSEN2* mutations, the age at onset ranges from 40 to 85 years of age. In contrast, individuals belonging to families that develop Alzheimer's due to mutations in *APP* or *PSEN1* experience an age at disease onset between 35 to 55 (*PSEN1*) or 45 to 65 years of age (*APP*).

## 3. Late Onset Alzheimer's Disease

 In contrast to FAD, which is inherited in a Mendelian dominant fashion as described earlier, sporadic or late onset Alzheimer's disease (LOAD) is etiologically heterogeneous and results from a combination of many genetic and environmental factors. Although there is evidence of a strong genetic component to disease risk, a large percentage of the genetic risk remains unidentified. The heritability of LOAD was estimated to be 79% in a large population-based study of Swedish twins [51]. However, until quite recently, only a single gene had been reliably associated with increased risk for Alzheimer's disease. The epsilon allele in the apolipoprotein E gene (*APOE*4) has been associated with substantially increased risk for LOAD as well as an earlier age of disease onset [52, 53]. A large number of additional polymorphisms have been associated with risk for LOAD, but replication of these associations has been inconsistent. 

 A search for “Alzheimer's” in the Gene Card database yields 1890 hits. These polymorphisms are located in genes regulating inflammation, oxidative stress, vascular biology, and protease function, among others (a complete list is available at http://www.alzgene.org/). It is likely that the failure to universally reproduce these associations is due at least in part to incomplete phenotypic characterization of study participants, inadequate samples sizes, and incomplete penetrance of risk alleles [54, 55]. 

## 4. Genome-Wide Association Studies

 Although nearly 2000 genes have been implicated in the etiology of late onset Alzheimer's disease over the past two decades, it was only in 2009 that two large-scale genome-wide association studies replicated associations between LOAD and genes other than *APOE* (Table 2). In these studies, which were published back to back in Nature Genetics [56, 57], Lambert et al. and Harold and colleagues described associations between late onset Alzheimer's disease and genetic markers in three genes in addition to *APOE*: clusterin (*CLU*), complement receptor 1 (*CR1*), and phosphatidylinositol binding clathrin assembly protein (*PICALM*). In a study supported by the Welcome Trust and based in Cardiff, UK, Harold et al. employed a two-stage design with 3,941 Alzheimer's cases and 7,948 controls in the first stage, followed by genotyping of the most interesting variants in 2,023 cases and 2,340 controls. Lambert and colleagues conducted a similarly large study based at the Institute Pasteur in Lille, France of 2,032 cases and 5,328 controls, which replicated a European sample of 3,978 Alzheimer's cases and 3,297 controls. These studies were followed in 2010 by a meta-analysis of over 35,000 participants, including 8,371 individuals diagnosed with Alzheimer's disease [58]. In this meta-analysis, Seshadri and colleagues confirmed associations with *APOE*, *CLU*, *PICALM*, and *CR1* and identified two new Alzheimer's risk loci: Myc box-dependent-interacting protein 1 (*BIN1*) and a marker (rs597668) near the *EXOC3L2*/*BLOC1S3*/*MARK4 *gene cluster. 

 It is interesting that *BIN1* showed a nonsignificant trend for association in the previous studies by Harold et al. and Lambert et al. The significant signal for *BIN1* in the meta-analysis after a nonsignificant trend for association in the two initial GWAS studies was a clear indication of the tremendous sample sizes that are required to detect Alzheimer's risk genes outside of *APOE*. Current studies underway through the International Genomics of Alzheimer's Project (IGAP) will ultimately evaluate over 60,000 participants including more than 30,000 Alzheimer's cases, roughly twice the size of the previous largest effort by Seshadri et al. [58]. The IGAP consortium was launched in February 2011 as a collaborative effort between the European Alzheimer's Disease Initiative (EADI) from France, the Alzheimer's Disease Genetics Consortium (ADGC) and the neurology subgroup of the Cohorts for Heart and Aging in Genomic Epidemiology (CHARGE) from the United States, and the Genetic and Environmental Risk in Alzheimer's Disease (GERAD) from the United Kingdom. Data and DNA will be contributed by more than 35 individual institutions from Europe and North America. 

## 5. Copy Number Variation (CNV)

 With the advent of whole-genome scanning methods, a new perspective on human genetic variation was observed, the widespread variation in the copy number of submicroscopic DNA segments. CNVs offer an alternate genetic marker map for disease association studies. CNVs are major contributors to genetic variance; thus, it is conceivable that they may confer susceptibility to or directly cause disease [59]. CNVs influence gene expression, phenotypic variation and adaptation by altering gene dosage [59]. A recent study of gene expression variation as a model of complex phenotypes found that 18% of gene expression traits were associated with CNVs [60]. With regard to Alzheimer's disease, Brouwers et al. reported a significant association between CNVs in the CR1 gene and increased risk for Alzheimer's [61]. In this study, a Flanders-Belgian cohort of 1,887 individuals was used as a discovery set combined with a French validation cohort of 2,003 individuals. CNV within the gene was observed to produce two CR1 isoforms. One of these was associated with increased Alzheimer's risk (*P* = 0.0025 for the combined cohorts). The authors hypothesized that the association with the high copy number variant was caused by additional C3b/C4b and cofactor sites [61]. In addition, Szigeti and colleagues detected significant association between a high copy number segment on chromosome 14 (14q11.2) in the region of the olfactory receptor and increased risk for Alzheimer's disease. This finding is of interest due to the early involvement of the olfactory lobes in Alzheimer's neuropathology [62, 63].

## 6. LOAD Genes

### 6.1. Apolipoprotein E (APOE)

The APOE protein is 299 amino acids long and is synthesized principally in the liver. In the nervous system, nonneuronal cells (astroglia and microglia) are the primary producers of APOE. It transports lipoproteins, fat-soluble vitamins, and cholesterol into the lymphatic system and then into the blood. APOE is the ligand for the LDL receptor, LRP (LDL-related protein), and VLDL-receptor, all of which are preferentially expressed by neurons. Given its involvement in lipid transport, it is perhaps not surprising that the role for *APOE* as a disease gene was first recognized with respect to cardiovascular disease [64–66].

The *APOE* gene is located on chromosome 19q13.2 in a cluster with apolipoprotein C1 and apolipoprotein C2. The gene is on the small side, containing 3,597 nucleotides and four exons [67]. Three primary isoforms exist for apolipoprotein E (E2, E3, and E4), which are encoded by 3 alleles (epsilon 2, 3, and 4). These isoforms differ in the amino acid sequence at 2 sites: residues 112 and 158. At these sites, the E2, E3, and E4 isoforms contain cysteine/cysteine, cysteine/arginine, and arginine/arginine residues, respectively [67–69]. The absolute allele frequencies vary widely by race and ethnicity [70–72], with the E4 allele showing a curvilinear relationship to latitude, with lowest frequency in the mid-latitudes [73]. Eisenberg and colleagues, who reported this relationship, have suggested that it is due to a reduced metabolic rate and hence lowers cholesterol requirements in more moderate climates [73].

 The effect size for the *APOE*4 allele is among the largest for any multifactorial, oligogenic (complex) disease. Individuals homozygous for *APOE*4 are 10 to 20 times as likely to develop LOAD as *APOE*4 negative individuals [74, 75], and there is a gene-dosage effect observed between increasing copies of the E4 allele and earlier age of disease onset [76]. In contrast, individuals who carry the *APOE*2 allele have a lower risk for Alzheimer's disease relative to other genotypes [77]. However, LOAD is clearly a heterogeneous disease, as one third of Alzheimer's patients are *APOE*4 negative. Interestingly, health risks associated with carriage of the *APOE*4 allele are not specific to Alzheimer's disease. A worse outcome has been reported for *APOE*4 carriers following traumatic brain injury [78], selective cardiac bypass surgery [79], and spontaneous intracerebral hemorrhage [80].

 Even though the *APOE*4 allele has one of the greatest effect sizes of any gene associated with an oligogenic disease, the polymorphism accounts for less than half the genetic variance in Alzheimer's disease risk, strongly suggesting that additional Alzheimer's disease genes remain to be identified. Data from several recent large GWAS studies support this contention [56, 57, 81, 82].

### 6.2. Clusterin (CLU)

Clusterin is a 75-kDa apolipoprotein that is widely expressed throughout the body, particularly in the brain [83]. Structurally, CLU is heterodimeric, comprised of two subunits that are linked by disulfide bonds [84]. These subunits are formed by proteolytic cleavage of the clusterin precursor protein into alpha- and beta-peptide fragments [83]. Clusterin contains two coiled-coil *α*-helices, which are typical of small heat shock proteins, and it has been suggested that clusterin is in fact a heat shock protein [85]. The *CLU* gene is located on chromosome 8 (8p21) and has high sequence homology (70%–80% identity) across mammalian taxa. The gene is composed of 9 exons, covering 16Kb of DNA [86]. The name clusterin is derived from an ability to cluster together cells of various types [87]. The former name of this glycoprotein is APOJ, and like its cousin APOE, clusterin has chaperone properties and is able to interact with many different molecules [88]. The similarity to APOE extends to an elevated abundance in brain regions (including the hippocampus and entorhinal cortex) that are affected by the pathology of Alzheimer's disease and cerebrospinal fluid (CSF), as well as being present in amyloid plaques and binding to beta-amyloid [89–94]. Furthermore, both APOE and clusterin have been implicated in clearance of beta-amyloid from neural tissue and CSF [95, 96]. Other identified functions for clusterin include the regulation of complement formation and apoptosis, as well as lipid transport [97]. However, many of these findings have received inconsistent support. The one function that is consistently supported for CLU is to act as a chaperone protein.

### 6.3. Phosphatidylinositol Binding Clathrin Assembly Protein (PICALM)

PICALM is a ubiquitously expressed 70 kDa protein that has been implicated in the membrane retrieval of the synaptic vesicle [98]. PICALM is distributed in pre- and postsynaptic structures [99] and functions in clathrin-mediated endocytosis (CME) [100]. The involvement of PICALM in CME is important, as this process is a critical step in the intracellular movement of lipids and proteins [99] as well as internalization of full-length APP from the cell surface in cell culture studies [101]. However, mice that possess nonsense point mutations in the *PICALM* gene have abnormal hematopoiesis and iron metabolism but do not exhibit abnormal neurological function [102]. The *PICALM* gene was first identified in studies of myelogenous leukemia as the fusion partner of AF10 in a chromosomal translocation that is observed in acute myeloid leukemia, acute lymphoblastic leukemia, and malignant lymphoma (10;11)(p13;q14) [103]. Meyerholz and colleagues observed that CALM associates with the alpha-appendage domain of the AP2 adaptor via the three peptide motifs 420DPF, 375DIF, and 489FESVF and to a lesser extent with the amino-terminal domain of the clathrin heavy chain [104]. The *PICALM* gene is located on chromosome 11 (11q14.2), and the first 289 amino acids of the protein have a high degree of homology (81%) to the clathrin assembly protein AP3 [105]. There are three isoforms; the canonical sequence is 652 amino acids in length. Two additional isoforms are generated by deletions of short sequences of amino acids near the 3 ends of the transcript.

### 6.4. Complement Receptor 1 (CR1)

Complement receptor 1 (also known as CD35, C3b/C4b receptor) is a member of the receptor of complement activation (RCA) family. The CR1 protein is a monomeric type I membrane glycoprotein that is expressed on erythrocytes, leukocytes, and splenic follicular dendritic cells [106]. CR1 is the human receptor for C3b and C4b complement cleavage fragments [107]. As such, CR1 serves as the main system for processing and clearance of complement opsonized immune complexes and mediates cellular binding to particles that are labeled with activated complement [106]. It has been shown that CR1 can act as a negative regulator of the complement cascade, mediate immune adherence and phagocytosis, and inhibit both the classic and alternative complement pathways [106]. The number of CR1 molecules decreases with aging of erythrocytes in normal individuals and is also decreased in pathological conditions such as systemic lupus erythematosus (SLE), HIV infection, some hemolytic anemias, and other conditions featuring immune complexes [108, 109]. The *CR1* gene is located within a complex of immunoregulatory genes on chromosome 1 (1q32), known as the regulator of complement activation (RCA) superfamily [110, 111]. The *CR1* gene appears to have undergone multiple duplication events. The CR1 glycoprotein exists as four allotypic variants of variable sizes [112]. The most common CR1 isoforms are the “F” and “S” allotypes, which are 250 and 290 kDa, respectively. The size difference is due to the inclusion of a long homologous repeat of 40–50 kDa [112]. Certain alleles of this gene have been statistically associated with an increased risk of developing late onset Alzheimer's disease [56]. 

### 6.5. Bridging Integrator 1 (BIN1)

 BIN1, which was originally known as Myc box-dependent-interacting protein 1, is a widely expressed protein that interacts with Myc-box regions of the MYC oncoprotein [113]. Structurally, the terminal portion of BIN1 is related to amphiphysin, a cancer-associated autoantigen, and to RVS167, a regulator of the cell cycle in yeast [113]. Based on these observations, Sakamuro and colleagues concluded that BIN1 acts as a tumor suppressor through negative control of the cell cycle. BIN1 is a 70 kDa nuclear protein, as determined by immunoprecipitation and immunofluorescence experiments [113]. The *BIN1* gene is located on chromosome 2 (2q14.3). The gene spans 59.3 Kb and contains up to 20 exons [114]. As many as 10 isoforms of BIN1 exist, and these are produced by variable splicing of the mRNA [114]. The largest of these is isoform one, which is expressed exclusively in the brain and concentrated in nerve terminals (NCBI GeneID 274; http://www.ncbi.nlm.nih.gov/sites/entrez?db=gene&cmd=Retrieve&dopt=full_report&list_uids=274). The other smaller isoforms are generated by deletion of downstream exons, particularly 7, 11, 13, and 14 (NCBI GeneID 274).

### 6.6. ATP Binding Cassette Transporter 7 (ABCA7)

 ABCA7 is a 2,146-amino acid protein containing two highly conserved ATP binding cassettes [115]. The gene was first identified in macrophages and is expressed abundantly in myeloid cells, particularly monocytes and granulocytes [115]. Expression is induced by differentiation of monocytes into macrophages [115]. Like other members of the ABC family, ABCA7 expression shows a response to changes in lipid concentration. In macrophages, both mRNA and protein are upregulated in the presence of modified low-density lipoprotein and downregulated by HDL [115]. The ABCA7 gene is located on chromosome 19p13.3 and contains 46 exons and spans about 32 kb [115, 116]. The mRNA is 6.8 kb in length and encodes a polypeptide of 2146 amino acids with a calculated molecular weight of 220 kDa [115].

### 6.7. Membrane-Spanning 4-Domains, Subfamily A (MS4A)

 Three members of this family (MS4A4A, MS4A4E, and MS4A6E) were associated with AD by GWAS analysis [81, 117]. All three of these genes are located on chromosome 11q12.2 [118, 119]. The linkage disequilibrium and genomic structure in the region precludes assignment of the GWAS signal to a precise gene. The MS4A gene family is comprised of 16 genes clustered in a 600 kb region of chromosome 11q12 [119]. Most members of the *MS4A* gene family encode proteins with four or more transmembrane domains and have cytoplasmic domains at the amino and carboxyl termini, which are typically encoded by distinct exons. The structure and presumed function of the MS4A genes are similar to CD20, the high-affinity IgE receptor beta chain [120]. Ishibashi and colleagues described an *MS4A4A* mRNA that was predicted to encode a 205-amino acid protein with a conserved phosphorylation site at the intracellular loop [118]. In contrast, Liang and Tedder identified an *MS4A4A* mRNA that encoded a predicted peptide with 220 amino acids [120]. The same group also predicted that MS4A4E was 220 amino acids in length and was 76% identical to MS4A4A, sharing a high degree of homology with the transmembrane as well as both intracellular domains [119]. Liang and colleagues determined that the *MS4A4E* gene contained seven exons and spanned more than 23 kb. In contrast to *MS4A4A* and *MS4A4E*, as well as most other *MS4A* family members, *MS4A6E* encodes a protein with only two transmembrane domains and no carboxyl terminal cytoplasmic domain. As expected, since it contains fewer domains, the MS4A6E protein is smaller at 147 amino acids in length. Liang et al. predicted that MS4A6E contained four exons and spanned only 5 kb.

### 6.8. Ephrin Type-A Receptor 1 (EPHA1)

 The EPH family is one of the largest of the receptor tyrosine kinase families, and members play crucial roles during development, particularly of the nervous system [121, 122]. Several EPH family members have also been implicated in oncogenesis [121]. The family is split into two groups, based upon the nature of the ligand. EPHA receptors bind to GPI-anchored ephrin-A ligands, while EPHB receptors bind to ephrin-B proteins that have a transmembrane and cytoplasmic domain [123]. EPH and ephrin signaling are important for the formation of segmented structures [124] and for the control of axon guidance during development [125]. The EPHA1 protein contains 976 amino acids and is approximately 108 kDa [126]. The *EPHA1* gene is located on chromosome 7q34 and contains 18 exons that span a little over 18 kb [127]. 

### 6.9. CD 33 Antigen (CD33)

 CD33 belongs to a family of cell-surface receptors that are expressed in the peripheral circulation on monocytes and myeloid progenitor cells [128–130]. CD33 is a member of the immunoglobulin superfamily and functions as an adhesion molecule that mediates sialic acid-dependent binding to cells [131, 132]. The protein contains two immunoglobulin-like domains, a transmembrane region and a cytoplasmic tail that has two potential ITIM sequences [131]. CD33 may function as an inhibitory receptor by colligation with CD64 on myeloid cells [133]. *CD33* is located on chromosome 19q13.3 [127]. The gene contains seven exons that span 14.2 kb [134]. Alternate splicing of the transcript has been shown to result in two mRNA species of 1.4-1.5 kb and 1.6–1.8 kb [135]. The CD33 protein is 364 amino acids in length and has a mass of approximately 40 kDa [136].

### 6.10. CD2 Associated Protein (CD2AP)

 CD2AP is a docking protein that becomes tyrosine phosphorylated in response to extracellular stimuli such as growth factors or cell-cell interaction and subsequently induces vesicle formation [137]. Ligand binding of CD2AP triggers protein segregation, CD2 clustering, and cytoskeletal polarization [138]. Kim and colleagues identified a CD2AP mutation in the splice acceptor region of exon 7 that was associated with primary focal segmental glomerulosclerosis [139]. There was no stable protein transcribed from the variant allele, suggesting that haploinsufficiency of CD2AP caused the disorder. The protein comprises 639 amino acids and has a deduced molecular mass of approximately 70 kDa [137]. The gene is ubiquitously expressed in adult and fetal human tissues as an approximately 5.4 kb transcript [137]. 

### 6.11. Sortilin-Related Receptor 1 (SORL1)

*SORL1* encodes a protein that acts as a cell-surface receptor that binds ApoE and assists in intracellular trafficking of APP [140]. There is also evidence that SORL1 is important for the processing of APP by presenilins and the production of beta-amyloid [141]. The gene is located on chromosome 11q24.1 and encodes a 2,186-amino acid polypeptide that has homology to the RAP binding receptor gp95/sortilin [142]. Support for *SORL1* as an Alzheimer's disease risk gene has been mixed, but a recent meta-analysis of previous studies detected a significant association between clusters of polymorphisms in *SORL1* and Alzheimer's disease in both Caucasians and Asians [143]. These results, in combination with significant associations between *SORL1* polymorphisms and hippocampal atrophy [144], as well as CSF levels of beta amyloid 42 [145], have increased confidence in the gene as an AD candidate.

## 7. Pathway Analysis

Evidence of the need for additional genetic research into Alzheimer's disease is provided by the fact that despite intensive searching over the past two decades, roughly half of the predicted genetic variation in Alzheimer's disease risk has been identified. Previous efforts have involved family-based linkage studies, population-based genome-wide association studies, and a host of candidate gene association tests. Recently, GWAS methods have been extended to very large numbers of participants. While it is true that one way to identify Alzheimer's genes with vanishingly small effect sizes is to employ ever-increasing numbers of participants in collaborative studies and meta-analyses, this approach relies upon the detection of marginal effects of SNPs within a single haplotype block. Even though sample sizes for recent GWAS studies are in the tens of thousands of participants, which supply an impressive amount of statistical power, this approach remains largely unable to resolve gene-gene interactions, which critically underlie the common gene-common disease hypothesis. Detection of multiple interacting loci requires a more sophisticated analytical approach.

 One method for detecting interacting alleles within biological pathways is network enrichment analysis, which has also been termed pathway analysis [146]. This technique was originally developed for the analysis of microarrayed gene expression data [146]. The basis of this analytical method is to identify biological pathways, rather than individual markers or genes that are associated with the outcome of interest [147]. First, standard GWAS data are generated. Next, a gene assignment is made for as many markers as possible, and an adjustment is made for the number of markers per gene. Then, genes are assigned to predefined biological pathways, using databases such as the Kyoto Encyclopedia of Genes and Genomes (KEGG) or the Gene Otology (GO) network. Finally, pathways or networks are evaluated for a significant overrepresentation of markers associated with the outcome of interest, relative to what would be expected at random. 

 One of the first applications of pathway analysis to Alzheimer's disease was published by Lambert et al. in 2010 [148]. In this study, Alligator and GenGen/KEGG software packages were used to analyze GWAS data derived from 2,032 Alzheimer's cases and 5,328 controls of French Caucasian ancestry. Both enrichment approaches identified a role for immunological dysfunction in the development of Alzheimer's disease [148]. While this association was far from novel, confirmation of a role for inflammation via pathway analysis supports the utility and value of pathway analysis methods in the study of Alzheimer's disease. In a second study from the same group in the same year, Hong and colleagues reported the involvement of intracellular transmembrane protein transport in Alzheimer's [149]. Of interest in the Hong et al. study was evidence of a functional role for *TOMM40* in the development of AD. *TOMM40* is the channel-forming subunit of the mitochondrial outer membrane translocase complex. Variation in the length of a poly-T homopolymer in this gene has been implicated in Alzheimer's etiology [150–154]; however, the validity of this association has been controversial due to strong linkage disequilibrium between *TOMM40* and *APOE*, which complicates interpretation of the signal [150, 155, 156]. In a more recent pathway analysis of GWAS data from 742 participants enrolled in the Alzheimer's Disease Neuroimaging Initiative (ADNI) project, Ramanan et al. identified a number of genes, pathways and networks [157]. In this study, the outcome variable was not disease status, but rather a composite memory score that was constructed from the ADNI neuropsychological battery. Using the GSA-SNP software tool, 27 canonical pathways were identified that were over enriched relative to the composite memory score [157]. Among the set of enriched pathways were biological processes known to be related to memory consolidation such as receptor-mediated calcium signaling and long-term potentiation. Additional processes that were enriched against the composite memory score included cell adhesion and neuronal differentiation, as well as glucose signaling and inflammation. Furthermore, a large gene set was identified with MetaCore software that was centered on SP1 transcriptional regulation [157]. 

 Pathway enrichment analysis has only recently been applied to Alzheimer's disease and most of these studies have been conducted in what are now relatively small cohorts. It is likely that as GWAS data are developed for large international cohorts, greater insight will be gained from interrogation of these data by pathway enrichment techniques. The combination of large sample sizes and sophisticated analytical methods such as pathway enrichment analysis is likely to produce many novel targets for the treatment and therapy of Alzheimer's disease.

## 8. Pharmacogenetics

Antidementia drugs are metabolized by the cytochrome p450 (*CYP*) gene family [158, 159]. Several of the *CYP* genes are highly polymorphic, particularly *CYP2D6*, *CYP2C19*, *CYP2C9*, and *CYP3A4/5*, and allelic frequencies at these loci vary greatly by ethnicity [158]. This is of concern since these allelic variants have substantial functional consequences, which places individuals who are slow metabolizers at risk of severe adverse events. Only 25% of western populations are rapid metabolizers, which places the remaining 75% of individuals who are prescribed antidementia drugs at risk due to overdosing [158]. The proper administration of antidementia drugs that are metabolized by *CYP* genes is to initially prescribe a low dose and titrate it upwards to the maximum tolerable dose. This presents another concern, in that if the starting dose is not escalated, the maximum effective dose may not be reached. Several excellent reviews of this literature have been provided by Cacabelos and colleagues [158, 160–162].

## 9. Summary

 Alzheimer's disease is a major health problem globally, with massive human and economic costs. Alzheimer's has been one of the most difficult diseases to defeat, and there are currently no proven effective means of cure or prevention. The genetic causes run the entire range from a Mendelian dominant transmission in FAD to risk factors for a complex multifactorial and etiologically heterogeneous disease in LOAD. In addition, a number of genetic polymorphisms are known to impact response to ant-dementia medication through pharmacogenetic effects. While many (perhaps most) causal alleles have been identified for FAD, only roughly half of the genetic variation for LOAD has been reliably identified. Future work toward discovering this missing heritability will likely involve studies of epigenetic phenomena, such as methylation and acetylation, as well as the control of gene expression by micro-RNA species. The advent of genome-wide methods has led to the identification of several risk loci for LOAD, in addition to the well-documented association with the APOE4 allele. However, there is a great need for further study. The advent of genome-wide scanning methods including affordable whole genome sequencing, the assessment of epigenetic mechanisms, and the development of more sophisticated statistical analysis methods will facilitate the identification of additional risk loci for LOAD and lead to the development of effective treatment and prevention strategies for this devastating disease.

## Figures and Tables

**Table 1:** Genes responsible for early onset Alzheimer's disease, including chromosomal location and function.

Gene name (symbol)	Chromosomal location	Function of encoded protein
Amyloid precursor protein (*APP*)	21q21.3	Implicated in neuronal development and synaptic formation and repair
Presenilin one (*PSEN1*)	14q24.2	Cleavage of the amyloid precursor protein and NOTCH receptor proteins via overlapping, but distinct mechanisms [12, 13]
Presenilin two (*PSEN2*)	1q42.13	

**Table 2:** Genes reliably implicated in risk for late-onset Alzheimer's disease, including chromosomal location and function.

Gene name (symbol)	Chromosomal location	Function of encoded protein
Apolipoprotein E (*APOE*)	19q13.2	Transportation of lipoproteins, fat-soluble vitamins, and cholesterol
Clusterin (*CLU*)	8p21	Chaperone protein
Complement receptor 1 (*CR1*)	1q32	Receptor for C3b and C4b complement cleavage fragments, the main system for clearance of complement opsonized immune complexes
Phosphatidylinositol binding clathrin assembly protein (*PICALM*)	11q14.2	Membrane retrieval of the synaptic vesicle, intracellular movement of lipids and proteins, and possibly internalization of full length APP from the cell surface
Myc box-dependent-interacting protein 1 (*BIN1*)	2q14.3	Tumor suppressor
ATP binding cassette transporter 7* (ABCA7) *	19p13.3	The expression pattern suggests a role for lipid homeostasis and differentiation of immune cells.
Membrane-spanning 4-domains, subfamily A *(MS4A) *	11q12.2	Possibly involved in signal transduction or immunological functions
Ephrin type-A receptor 1 (*EPHA1) *	7q34	Member of the EPH receptor-tyrosine kinase family, implicated in mediating developmental events of the nervous system
CD33 antigen *(CD33) *	19q13.3	Adhesion molecule of myelomonocytic-derived cells
CD2 associated protein* (CD2AP) *	6p12.3	A scaffolding molecule that regulates the actin cytoskeleton and vesicle formation
Sortilin-related receptor 1* (SORL1) *	11q24.1	Receptor for ApoE, assists with intracellular trafficking and processing of APP
